# Histone H3 lysine 9 trimethylation is required for suppressing the expression of an embryonically activated retrotransposon in *Xenopus laevis*

**DOI:** 10.1038/srep14236

**Published:** 2015-09-21

**Authors:** Sarah Herberg, Angela Simeone, Mami Oikawa, Jerome Jullien, Charles R Bradshaw, Marta Teperek, John Gurdon, Kei Miyamoto

**Affiliations:** 1 Wellcome Trust/Cancer Research UK Gurdon Institute, University of Cambridge, Tennis Court Road, Cambridge CB2 1QN, UK

## Abstract

Transposable elements in the genome are generally silenced in differentiated somatic cells. However, increasing evidence indicates that some of them are actively transcribed in early embryos and the proper regulation of retrotransposon expression is essential for normal development. Although their developmentally regulated expression has been shown, the mechanisms controlling retrotransposon expression in early embryos are still not well understood. Here, we observe a dynamic expression pattern of retrotransposons with three out of ten examined retrotransposons (*1a11, λ-olt 2-1* and *xretpos(L)*) being transcribed solely during early embryonic development. We also identified a transcript that contains the long terminal repeat (LTR) of *λ-olt 2-1* and shows a similar expression pattern to *λ-olt 2-1* in early *Xenopus* embryos. All three retrotransposons are transcribed by RNA polymerase II. Although their expression levels decline during development, the LTRs are marked by histone H3 lysine 4 trimethylation. Furthermore, retrotransposons, especially *λ-olt 2-1*, are enriched with histone H3 lysine 9 trimethylation (H3K9me3) when their expression is repressed. Overexpression of lysine-specific demethylase 4d removes H3K9me3 marks from *Xenopus* embryos and inhibits the repression of *λ-olt 2-1* after gastrulation. Thus, our study shows that H3K9me3 is important for silencing the developmentally regulated retrotransposon in *Xenopus laevis*.

Transposons are mobile DNA elements that have the ability to propagate or translocate in the genome. Retrotransposons use a reverse-transcribed RNA intermediate for their transposition. They can be divided into two major categories: retrotransposons with or without long terminal repeats (LTR- and non-LTR retrotransposons, respectively). For example, after transcription of LTR retrotransposons the produced RNA is reverse-transcribed, leading to the formation of a double-stranded DNA including complete LTRs. This DNA can then be reintegrated into the genome^1^. In order to protect the integrity of the genome, transposable elements (TEs) ought to be silenced in cells. The transcription of many TEs is repressed by DNA methylation or by repressive histone modifications, such as histone H3 lysine 9 trimethylation (H3K9me3)^1^. TEs are also silenced post-transcriptionally, for example by RNA interference^2^ or by piwi-interacting RNAs in germ line cells^3^.

However, various findings have shown that TEs are not completely silenced in early mouse embryos^4^,^5^,^6^. Between 15–20% of the early embryonic mouse transcriptome consists of repetitive elements, with LTR retrotransposons representing 33–49% of these repetitive elements^6^. Furthermore, transposition of the human or mouse L1 retrotransposons was found to occur in transgenic mice and rats during embryogenesis, leading to somatic mosaicism^5^. Additionally, LTRs of retrotransposons can function as alternative promoters and first exons during mouse embryogenesis^4^, forming chimeric transcripts of retrotransposons and protein coding genes. Chimeric transcripts formed by this means are not expressed at the same developmental stages as their conventional non-chimeric counterparts^4^, suggesting that retrotransposons can control developmentally regulated expression of these transcripts.

Some studies found that the expression of retrotransposons in early mouse embryos is important for embryonic development. For example, murine endogenous retrovirus-like (MuERV-L), a mouse LTR retrotransposon that is transcriptionally activated 8–10 h after fertilization, is needed for the development of mouse embryos to the 4-cell stage^7^. Moreover, MuERV-L was found to form chimeric transcripts in mouse embryos^4^ and marks the sub-population of embryonic stem cells, which carry the property of 2-cell stage embryos^8^. Another retrotransposon that seems to be important for the embryonic development of mice is LINE-1. The microinjection of morpholino antisense oligonucleotides targeting LINE-1 irreversibly arrests embryonic development of mice^9^. Since the expression of specific TEs in early embryos is essential for normal development, it is important to understand the mechanisms leading to TE activation in early embryonic stages and subsequent TE silencing. DNA demethylation does not seem to play a major role in TE activation in mouse embryos^10^. Instead, histone modifications were shown to regulate expression of TEs^1^. In mouse embryos, retrotransposons are marked by the repressive histone mark H3K9me3 and by the activating histone mark histone H3 lysine 4 trimethylation (H3K4me3), which is lost in parallel to transcriptional repression of retrotransposons^6^.

In contrast to regulation of retrotransposons in mammalian embryos, almost nothing is known about epigenetic mechanisms of retrotransposon regulation in *Xenopus* embryos. It has been shown that *Xenopus* has very few bivalent domains marked by both H3K4me3 and histone H3 lysine 27 trimethylation (H3K27me3) to regulate expression of developmentally important genes, being different from mammals and zebrafish embryonic chromatin^11^,^12^. Therefore, it is possible that different regulation mechanisms of embryonically active retrotransposons may exist in *Xenopus*. Moreover, *Xenopus* embryos allow the collection of a large number of *in vivo* embryonic cells when zyogotic transcription starts. In gene expression database several retrotransposon transcripts of *Xenopus laevis* could be identified. In addition, the expression of a few retrotransposons was reported to be developmentally regulated^13^,^14^. Based on this knowledge, we further investigated the expression of retrotransposons in *Xenopus laevis* and found retrotransposons whose expression is upregulated in early embryos. These retrotransposons are marked by H3K4me3 and H3K9me3 on their LTRs at different embryonic stages. Enzymatic removal of H3K9me3 marks from *Xenopus* embryos leads to the enhanced expression of a specific retrotransposon, demonstrating a role of H3K9me3 in the transcriptional repression of the developmentally regulated retrotransposon.

## Results

### The transcript levels of the retrotransposons *1a11, λ-olt 2-1 and xretpos(L)* are upregulated after midblastula transition and downregulated after the gastrula stage

**Figure 1:**
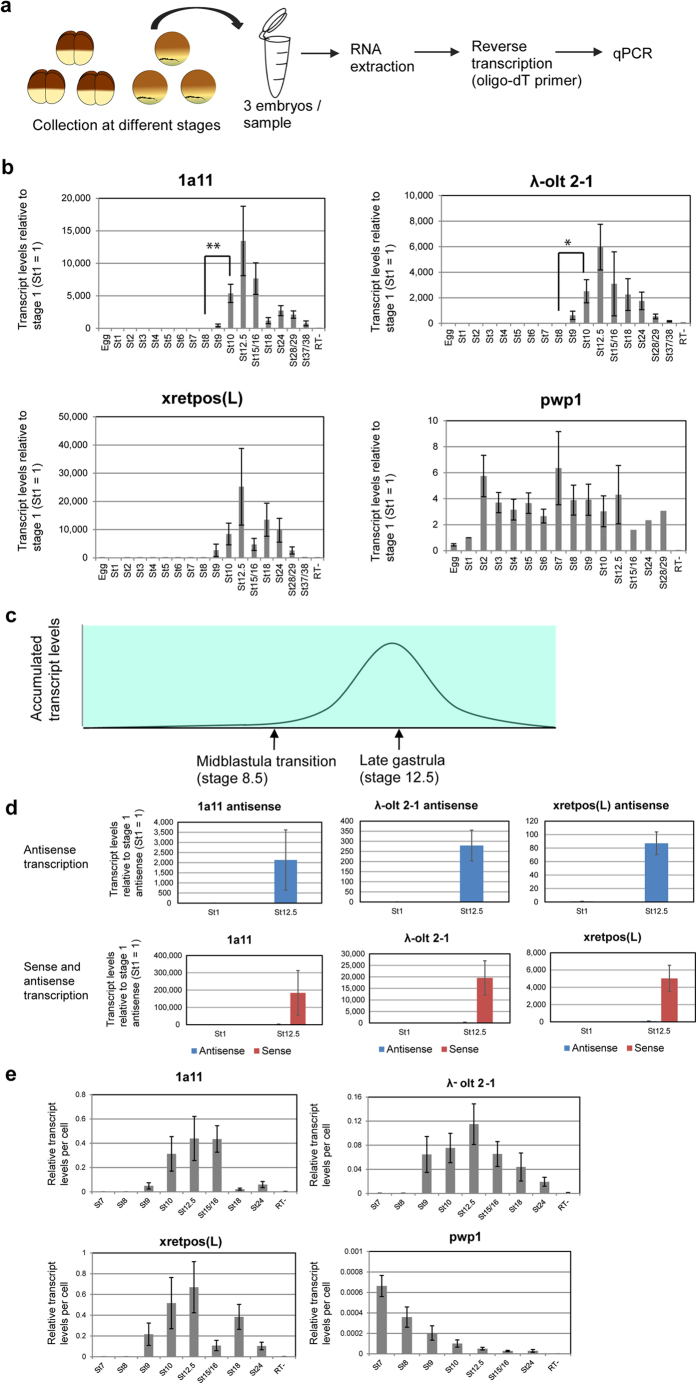
Developmentally regulated expression of retrotransposons *1a11, λ-olt 2-1* and *xretpos(L)* during *Xenopus laevis* embryonic development. (**a**) Schematic diagram of expression analysis during embryonic development. Embryos were collected at different stages. After RNA extraction and reverse transcription the transcript level was determined by qPCR. The container was drawn by S.H. (**b**) Relative changes of the transcript levels of *1a11, λ-olt 2-1* and *xretpos(L)* during embryogenesis in comparison to their transcript levels at the one-cell stage (St1 = 1) (n = 3–14). *pwp1* is a constantly expressed gene that was used as control (n = 1–4). All values were normalized to the overall RNA concentration of the respective sample. Sample RT- is a negative control. Error bars represent SEM. **P = 0.005, *P = 0.021. (**c**) Schematic diagram of the accumulated transcript levels of *1a11, λ-olt 2-1* and *xretpos(L)* during embryogenesis, explaining that their expression is upregulated after midblastula transition and shows the highest level at the late gastrula stage. (**d**) *1a11, λ-olt 2-1* and *xretpos(L)* are mainly detected as sense transcripts although antisense transcription can also be found. Forward primers were used for reverse transcription to analyze the expression of antisense transcripts from retrotransposons (blue bars), while reverse primers were used as a control (red bars). Upper graphs summarize antisense transcription at stages 1 and 12.5. Lower graphs represent the comparison between sense and antisense transcription although antisense transcription is almost invisible due to its much weaker expression than sense transcription. Relative transcript levels were compared to the transcript levels at the one-cell stage (St1 = 1) (n = 4). (**e**) Normalization by the cell number also indicates the upregulated expression of retrotransposons after midblastula transition and the downregulated expression after the gastrula stage. Relative changes of the transcript levels of *1a11, λ-olt 2-1, xretpos(L)* and *pwp1* per cell during embryogenesis in comparison to their transcript levels at the one-cell stage (St1 = 1) (n = 3–13). Error bars represent SEM. Only values from stage 7 onwards are shown, since the increase of the cell number is less constant during the first six stages (Supplementary Fig. S3).

To identify retrotransposons that are specifically activated during early embryonic development, we first performed RNA-sequencing (RNA-seq) analysis of gastrula embryos (stage 10.5–11.5) and calculated RPKM (reads per kilobase per million mapped reads) values of putative retrotransposons, whose transcript sequences are available in NCBI database (Supplementary Table S1). Their transcripts were detected in gastrula embryos (Supplementary Table S1). Among them, the expression of 10 different putative retrotransposons was examined in eggs and embryos (stage 1–37/38) by reverse transcription-quantitative polymerase chain reaction (RT-qPCR) (Fig. 1a,b, Supplementary Fig. S1). Using this method, we are able to determine overall transcript levels of retrotransposons existing in numerous copies at different genomic loci (see Discussion). Three retrotransposons were found, whose transcript levels are upregulated after the midblastula transition and downregulated after the gastrula stage (Fig. 1b,c). These three retrotransposons are *1a11*^13^ (GenBank accession number: L11263.1), *λ-olt 2-1*^14^ (GenBank accession number: AF145965.1) and *xretpos(L)*^14^ (GenBank accession number: AF057166.1). *λ-olt 2-1* and *xretpos(L)* show 95% nucleotide sequence identity in 1595 nucleotides^14^. These retrotransposons start to be activated soon after the midblastula transition. Then, the transcript levels of *1a11*, *λ-olt 2-1* and *xretpos(L)* in whole *Xenopus laevis* embryos increase from the blastula stage (stage 8) to the gastrula stage (stage 12) and gradually decrease after the late gastrula stage (stage 12.5) (Fig. 1b). For this analysis all values were normalized to the total RNA concentrations of each samples. Normalization to the transcript level of a constantly expressed control gene leads to similar results (Supplementary Fig. S2). Furthermore, we asked if these three retrotransposons are transcribed from both strands. Antisense transcription was detected at the gastrula stage (Fig. 1d, St12.5 at upper graphs), but the sense transcripts represent the vast majority of transcripts (Fig. 1d, red bars compared to almost invisible blue bars at lower graphs).

During embryonic development the cell number increases rapidly from one cell (stage 1) to 150,000 cells at the tail bud stage (stage 26) (Supplementary Fig. S3). Therefore, it is possible that the increase of the retrotransposon transcripts after stage 8 only reflects the rapid cell proliferation, whereas the transcript level per cell stays rather constant. In order to exclude this possibility, the transcript level per cell was calculated based on the total cell number estimation (Supplementary Fig. S3). These results also showed that the transcript level per cell increases after stage 8 and decreases after stage 12.5 (Fig. 1e). Thus it can be concluded that retrotransposons *1a11*, λ-*olt 2-1* and *xretpos(L)* show dynamic changes in transcription activities during embryonic development and the highest transcript levels of these are detected at the late gastrula stage.

### The LTR of *λ-olt 2-1* is found in a transcript, which contains a high homology sequence with *mars2*

**Figure 2:**
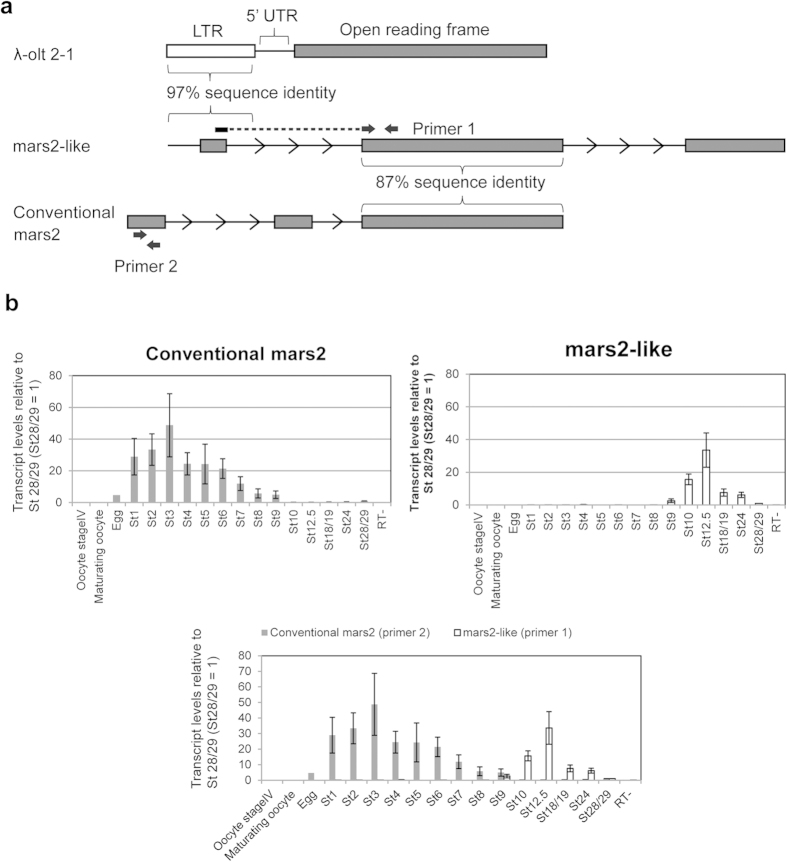
The differential expression pattern between *mars2-like* and conventional *mars2* during embryonic development. (**a**) The structure of *λ-olt 2-1*, *mars2-like* and conventional *mars2*. Grey boxes represent exons and the white box represents the LTR of *λ-olt 2-1*. The first exon of *mars2-like* and its surrounding region show 97% sequence identity to the LTR of *λ-olt 2-1*; the second exon shows 87% sequence identity to the third exon of conventional *Xenopus mars2*; the third exon shows no sequence identity to any reported gene. The full nucleotide sequence of *mars2-like* is shown in Supplementary Figure S5. Arrows represent binding sites of primer pairs used for qPCR. The forward primer of primer pair 1 binds to the splicing junction between exon 1 and exon 2 of *mars2-like*. Primer 2 binds to exon1 of conventional *mars2*. (**b**) Changes in the transcript levels of *mars2-like* and conventional *mars2* during embryogenesis in comparison to their transcript levels at stage 28/29 (St28/29 = 1). Stage 28/29 was used as a reference here instead of stage 1, since the transcript level of conventional *mars2* is already very high at stage 1. The figure at the bottom shows a merge of *mars2-like* (primer 1) and conventional *mars2* (primer 2). All values were normalized to the overall RNA concentration of the respective sample. Sample RT- is a negative control. All error bars represent SEM. n = 3–7.

It was previously shown that retrotransposons can regulate gene expression in early mouse embryos by functioning as alternative promoters and first exons^4^. Therefore, we asked if the three retrotransposons *1a11, λ-olt 2-1* and *xretpos(L)* also form chimeric (fusion) transcripts in early *Xenopus* embryos. To investigate this, a blast search for the three retrotransposons against a transcriptome assembly mapped to the *Xenopus laevis* genome 6.1^15^ was performed. The transcriptome was generated from the RNA-seq analysis of gastrula embryos (stage 10.5–11.5) using paired-end sequencing with Illumina HiSeq2000. We found a transcript consisting of three exons, with the first exon and its surrounding region showing 97% sequence identity to the LTR of *λ-olt 2-1* (Fig. 2a). The third exon shows no sequence identity to any reported gene. The second exon of this transcript, however, shows 87% sequence identity to the third exon of a gene called *mars2*. This *mars2* gene encodes a mitochondrial methionyl-tRNA synthetase, which is needed for the translation of mitochondrial genes. Interestingly, tRNA is involved in the transposition of retrotransposons^16^. Hereafter, the transcript containing the LTR of *λ-olt 2-1* as first exon (Fig. 2a, middle transcript) is referred to as *mars2-like*. The *mars2* transcript found under the NCBI Reference Sequence NM_001086369.1 (Fig. 2a, lower transcript) is referred to as conventional *mars2*. The splicing junction between *λ-olt 2-1* and *mars2-*high homology sequence was validated by continuous sequencing reads over the splicing junction (Supplementary Fig. S4). Moreover, we amplified *mars2-like* transcripts using primers located at the beginning of exon 1 and in exon 2, and sequenced the PCR product. This sequencing analysis further validated that *mars2-like* contains both retroelement *λ-olt 2-1* (exon1) and *mars2* homology sequence (exon 2) (Supplementary Fig. S5).

### *mars2* expression during embryonic development

Previous studies using mouse have shown that chimeric transcripts containing retrotransposons are specifically expressed in early embryos^4^. We therefore asked if the expression pattern of *mars2-like*, which contains the LTR of the retrotransposon, during embryonic development differs from that of the conventional *mars2*. To test this, different pairs of qPCR primers were designed to specifically detect either *mars2-like* or conventional *mars2* (Fig. 2a). The transcript level of conventional *mars2* (primer 2) is highest at the first three embryonic stages and then gradually decreases until the blastula stage (stage 9) (Fig. 2b). At this stage, the transcript level of *mars2-like* (primer 1) starts to increase (Fig. 2b). Interestingly, it seems that *mars2-like* transcript is substituting the transcript of conventional *mars2* (Fig. 2b, the bottom merged graph). The expression of *mars2-like* (primer 1) resembles the expression of *λ-olt 2-1* itself (Fig. 1b). These results suggest that *λ-olt 2-1* may regulate the expression of *mars2-like* transcript.

### *1a11, λ-olt 2-1, xretpos(L)* and *mars2-like* are newly transcribed by RNA polymerase II

**Figure 3:**
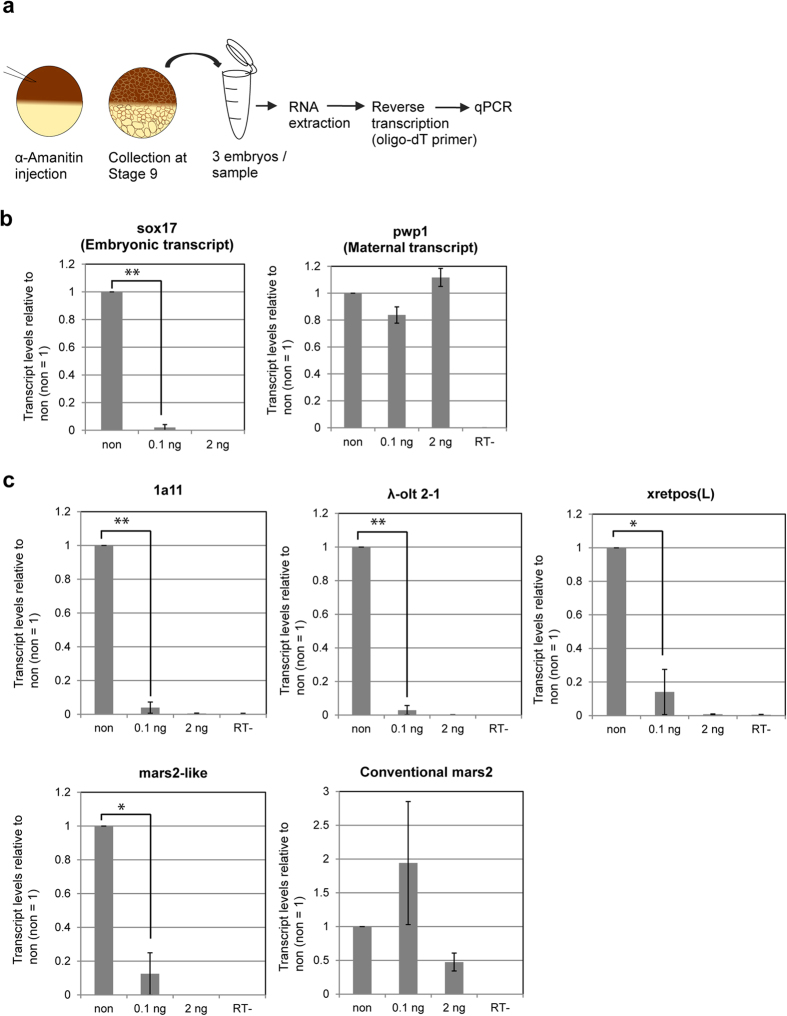
*1a11*, λ-o*lt 2-1, xretpos(L)* and *mars2-like* are transcribed by RNA polymerase II. (**a**) Schematic diagram of the transcriptional inhibition experiment. 0.1 ng or 2 ng of α-amanitin were injected into one-cell stage embryos. Embryos were collected at the blastula stage (stage 9) and RT-qPCR was performed. The container was drawn by S.H. (**b**) and (**c**) The effect of α-amanitin injection on the transcript levels of *sox17*, *pwp1*, *1a11, λ-olt 2-1, xretpos(L),* and *mars2-like*. Transcript level of non-injected embryos were used as a reference (non = 1). All values were normalized to the RNA concentration of the respective sample. Sample RT- is a negative control. All error bars represent SEM. n = 3. *P < 0.05, **P < 0.005.

Oocytes, direct precursors of eggs, contain some messenger RNAs (mRNAs) that have no or only very short poly(A) tails. During oocyte maturation and early embryonic development these short poly(A) tails are elongated, which facilitates translation^17^,^18^,^19^ and the amount of poly(A) tailed RNAs increase^20^,^21^. Since we were using oligo-dT primer for RT-qPCR (Fig. 1a), mRNAs without poly(A) tails or with too short poly(A) tails would not be reverse transcribed. If the transcripts of *1a11, λ-olt 2-1, xretpos(L)* and *mars2-like* had such short poly(A) tails in eggs, we would therefore not be able to detect them until these short poly(A) tails were elongated. Thus, it is possible that the low transcript level of *1a11, λ-olt 2-1, xretpos(L)* and *mars2-like* at the early embryonic stages is only an artifact, caused by the incapability of amplification by reverse transcription. In order to test if *1a11, λ-olt 2-1, xretpos(L)* and *mars2-like* are newly transcribed or if their transcripts undergo changes in lengthening their poly(A) tails, α-amanitin was injected into fertilized one-cell embryos at two different concentrations (2 ng and 0.1 ng) (Fig. 3a). At the lower concentration (0.1 ng injection) α-amanitin can be used as a specific inhibitor for RNA polymerase II, while the higher α-amanitin concentration such as 20 μg/ml also inhibits RNA Polymerase III^22^. Injected, normal-looking embryos were collected at the blastula stage (stage 9) and samples for qPCR were prepared (Fig. 3a). As a control, the transcript level of *sox17* and *pwp1* was measured. *sox17* expression, which is embryonically activated, was significantly inhibited by the addition of α-amanitin (P = 0.00002, Fig. 3b), indicating that α-amanitin treatment inhibits embryonic transcription. In contrast, the transcript level of *pwp1* was not influenced by the injection of α-amanitin (Fig. 3b), suggesting that *pwp1* transcripts are maternal transcripts. Even at the low α-amanitin concentrations the transcript levels of *1a11, λ-olt 2-1, xretpos(L)* and *mars2-like* were significantly (P < 0.05) reduced by the injection of α-amanitin (Fig. 3c). In contrast, the transcript level of conventional *mars2* does not seem to be influenced by the injection of α-amanitin. These results indicate that all three retrotransposons and *mars2-like* are newly transcribed by RNA polymerase II in embryos, while conventional *mars2* seems to be a maternal transcript.

### *1a11, λ-olt 2-1* and *xretpos(L)* are marked by H3K4me3 and H3K9me3

**Figure 4:**
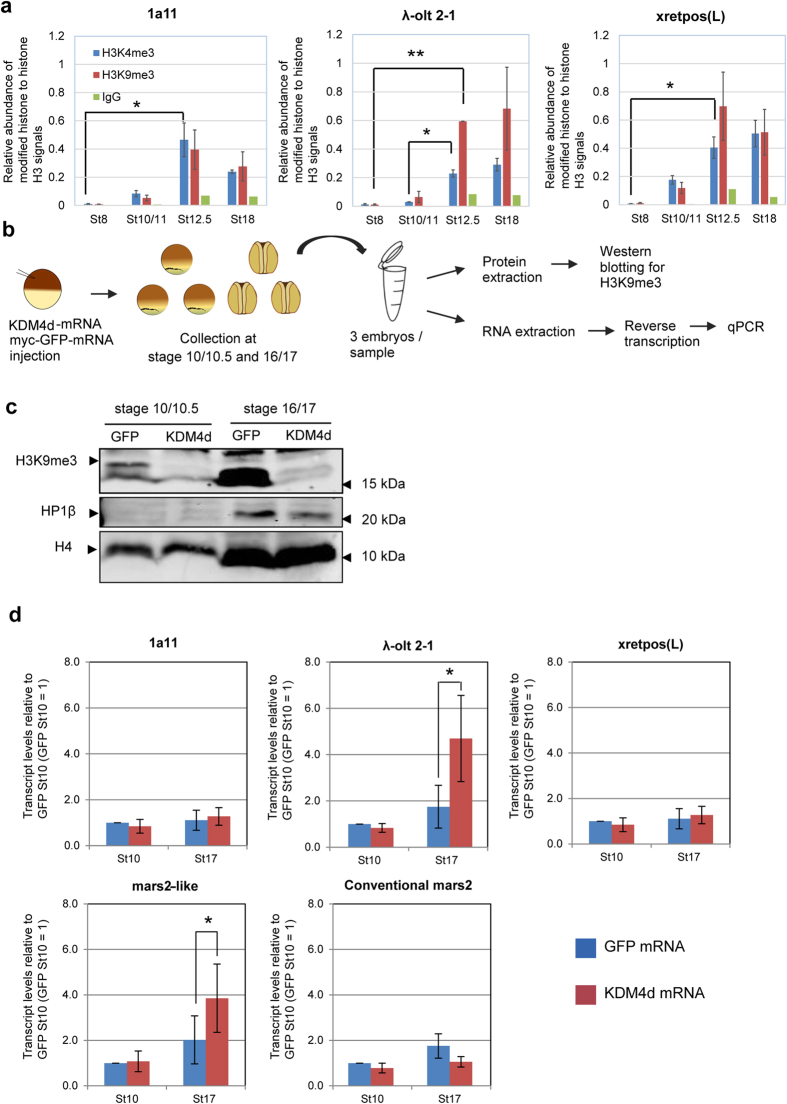
KDM4d overexpression removes H3K9me3 in embryos and upregulates *λ-olt 2-1* at the neurula stage. (**a**) ChIP analysis indicates enrichment of H3K4me3 and H3K9me3 on LTRs of retrotransposons at different stages of embryogenesis. The precipitated DNA/input DNA of *1a11, λ-olt 2-1* and *xretpos(L)* was determined by qPCR. (H3K4me3 and H3K9me3: n = 3–4, IgG: n = 2). All values of modified histone and control IgG were normalized to H3 (Supplementary Fig. 6b). All error bars represent SEM. **P = 0.00008, *P < 0.05. Blue = H3K4me3, red = H3K9me3, and green = IgG. (**b**) KDM4d mRNA was injected into fertilized embryos to remove H3K9me3 during embryogenesis. At the gastrula stage (stage 10) and at the neurula stage (stage 17) embryos were collected and prepared for western blotting or qPCR. Myc-GFP mRNA was used as a control. The container was drawn by S.H. (**c**) Removal of H3K9me3 by KDM4d mRNA injection was verified by western blot. The same samples were used to detect the expression of HP1β in *Xenopus* embryos. Histone 4 (H4) was used as a loading control. (**d**) The effect of KDM4d overexpression on retrotransposons expression and on the expression of *mars2-like* and conventional *mars2* at the gastrula (St10) and the neurula stages (St17). Transcription was measured by RT-qPCR. The relative change of the transcript level in comparison to the transcript level of control myc-GFP mRNA-injected embryos is shown. The transcript level of myc-GFP mRNA-injected embryos at stage 10 was set 1. All values were normalized to the RNA concentration of the sample. All error bars represent SEM. n = 3–5. *P < 0.05. Blue bars represent myc-GFP mRNA-injected embryos while red bars are KDM4d mRNA-injected embryos.

We next investigated mechanisms leading to the developmentally regulated expression of *1a11, λ-olt 2-1* and *xretpos(L)* transcription during embryogenesis (Fig. 1b, c). It was shown that retrotransposons in mouse embryos are marked by the active histone mark H3K4me3 and by the repressive histone mark H3K9me3^6^. Repression of those retrotransposons was accompanied by the loss of H3K4me3, rather than the gain of H3K9me3^6^. Therefore, we tested if LTRs of *1a11, λ-olt 2-1* and *xretpos(L)* are also marked by H3K4me3 and H3K9me3 before and after the gastrula stage, at which transcriptional levels of those retrotransposons become highest. We collected embryos at the blastula stage (stage 8), the early gastrula stage (stage 10/11), the late gastrula stage (stage 12.5) and the neurula stage (stage 16–18) to perform chromatin immunoprecipitation (ChIP) analysis. Non-specific rabbit immunoglobulin G (IgG) was used as a negative control and precipitated 0.15–0.006% of input DNA (Supplementary Fig. S6a) while histone H3 as a positive control precipitated up to 27% of input DNA (Supplementary Fig. S6b). Both H3K4me3 and H3K9me3 were enriched on LTRs of retrotransposons (Supplementary Fig. S6a). Relative enrichment of H3K4me3 to total histone H3 indicates that H3K4me3 levels increased up to stage 12.5 on all three retrotransposons (Fig. 4a, blue bars, *P < 0.05). A significant increase of H3K9me3 during developmental progression was observed in *λ-olt 2-1* (Fig. 4a, red bars, **P = 0.00008). These results suggest that the increase of H3K9me3 levels is correlated with transcriptional repression of retrotransposons and therefore might be important for the suppression of retrotransposon expression.

### KDM4d overexpression removes H3K9me3 and releases transcriptional repression of *λ-olt 2-1* at the neurula stage

Since the results of the ChIP analysis indicate that H3K9me3 is enriched on retrotransposons during embryonic development (Fig. 4a), we asked if this enrichment of H3K9me3 is required for retrotransposon repression in early and late embryonic stages. In order to reduce H3K9me3, mRNA of lysine-specific demethylase 4d (KDM4d), which specifically removes histone H3 lysine 9 trimethylation^23^,^24^, was injected into fertilized embryos. KDM4d mRNA-injected embryos were collected at the early gastrula (stage 10/10.5) and neurula (stage 16/17) stages and subjected to western blotting (Fig. 4b). The results showed that, in comparison to myc-GFP mRNA-injected control embryos, H3K9me3 was strongly reduced in KDM4d mRNA-injected embryos (Fig. 4c). Thus, H3K9me3 mark is removed in the gastrula and neurula embryos by KDM4d overexpression. Surprisingly, the removal of H3K9me3 mark did not cause any apparent phenotype in *Xenopus* embryos at least up to the neurula stage (stage 17).

Next, we asked if KDM4d overexpression influences the transcription of *1a11, λ-olt 2-1* and *xretpos(L)*. For this reason, KDM4d mRNA-injected embryos were used for qPCR (Fig. 4b). Neither the transcript level of *1a11* nor that of *xretpos(L)* was affected by the injection of KDM4d mRNA (Fig. 4d). However, interestingly *λ-olt 2-1* transcripts were significantly (P = 0.044) upregulated in KDM4d mRNA-injected embryos at the neurula stage (stage 17), but not at the early gastrula stage (stage 10) (Fig. 4d). This indicates that H3K9me3 removal by KDM4d overexpression is associated with derepression of *λ-olt 2-1* after gastrulation. KDM4d overexpression did not significantly affect the expression of other putative retrotransposons listed in Supplementary Table S1 (Supplementary Fig. S7), although Tx1L (AF027962.1)^25^ seems to be upregulated by the H3K9me3 removal.

Since *mars2-like* expression seems to be driven by *λ-olt 2-1* (Fig. 2), we also measured the transcript level of *mars2-like* in embryos depleted of H3K9me3. We found that the KDM4d mRNA injection significantly (P = 0.040) enhances *mars2-like* transcription in neurula embryos (stage 17) in good agreement with the transcriptional upregulation of *λ-olt 2-1* (Fig. 4d). In contrast, the expression of conventional *mars2* was not affected by KDM4d mRNA injection (Fig. 4d). These results suggest that *mars2-like* is also regulated by H3K9me3 and support the idea that *λ-olt 2-1* is involved in transcriptional regulation of *mars2-like*.

Even though H3K9me3 was also detected on *λ-olt 2-1* LTR at early gastrula stages (stage 10) (Fig. 4a), KDM4d mRNA injection only had an effect on the expression of *λ-olt 2-1* and *mars2-like* during the neurula stage (stage 17). A possible reason for this difference might be that some factors, which are associated with H3K9me3 and needed for heterochromatin establishment, are not yet expressed during gastrulation. These factors could for example be the Heterochromatin binding protein 1, which functions in heterochromatin mediated gene silencing and directly binds to H3K9me3^26^,^27^. By western blotting we showed that HP1β is not detectable in embryos at early gastrula stage (stage 10), but is expressed during neurula stages (stage 17) (Fig. 4c). It is therefore possible that the lack of HP1β during gastrulation may explain the insensitivity of *λ-olt 2-1* and *mars2-like* expression to H3K9me3 removal in gastrula embryos (Fig. 4d).

## Discussion

We examined the expression of ten putative retrotransposons during *Xenopus* embryogenesis and found that the expression of three of these retrotransposons (*1a11, λ-olt 2-1* and *xretpos(L)*) is upregulated after midblastula transition and downregulated after the gastrula stage. The LTR of *λ-olt 2-1* also forms a fusion transcript with a sequence that showed high similarity to *mars2.* All three retrotransposons are marked by the histone modifications H3K9me3 and H3K4me3. The KDM4d mRNA injection, which results in H3K9me3 removal, causes an increase of the *λ-olt 2-1* and *mars2-like* transcript levels at the neurula stage, indicating that H3K9me3 plays a role in the repression of *λ-olt 2-1* after gastrulation.

All three retrotransposons, *1a11, λ-olt 2-1* and *xretpos(L)*, have been found as LTR retrotransposons in *Xenopus laevis*^13^,^14^. *1a11* encodes a 71 kDa protein, which shows similarity to the viral structure protein Gag. It was estimated that the *Xenopus* genome contains hundreds of sequences related to the LTR of *1a11,* and 20–30 sequences related to its open reading frame^13^. The published expression analysis data also indicate transcriptional repression in late embryonic development, in good agreement with our qPCR data^13^ (Fig. 1b). The *Xenopus* genome seems to contain approximately 200 copies of *xretpos(L)* LTR, 15 copies of the *xretpos(L)* open reading frame, and 50 copies of the complete *xretpos(L)*^14^. Again, the published expression analysis data of *xretpos(L)* support our qPCR data^14^ (Fig. 1b). Moreover, it was shown by RNA and antisense RNA injections that *xretpos(L)* has a posterior-ventralizing activity during *Xenopus* embryogenesis^28^. All three retrotransposons have in common that they show no amino acid homology to the conserved *pol* gene. Since the *pol* gene encodes proteins needed for transposition (protease, reverse transcriptase, and integrase), it is likely that *1a11*, *λ-olt 2-1* and *xretpos(L)* have lost their ability to transpose^14^.

We found that the LTR of *λ-olt 2-1* can function as the first exon and forms a transcript with a sequence that has a high homology with *mars2* during *Xenopus* embryogenesis (Fig. 2a, called as *mars2-like*). However, we were not able to determine whether *λ-olt 2-1* is located near conventional *mars2* gene due to an incomplete understanding of *Xenopus* genome. The second exon of *mars2-like* transcript shows high nucleotide sequence homology to the third exon of conventional *mars2.* Conventional *mars2* encodes a mitochondrial methionyl-tRNA synthetase. Mutations of the human homologue of this gene cause a neurodegenerative disease called Autosomal Recessive Spastic Ataxia with Leukoencephalopathy (ARSAL). It is likely that retroelements-derived repetitive sequences surrounding human *Mars2* (e.g. Line 1 and Line2 elements) are mediating genomic rearrangements that cause ARSAL^29^. The methionyl-tRNA synthetase encoded by *mars2* is needed for the translation of mitochondrial genes. Interestingly, tRNAs are also used as primer for the reverse transcription of retrotransposons or retroviruses. Methionyl-tRNA for example functions as a primer of yeast and plant retrotransposons like Ty1, Ty2, Ty3, Ty5 and Ta1^16^. It is also suggested that *xretpos(L)* is using arginine-tRNA as primer for reverse transcription^28^. Therefore, the developmentally regulated *mars2* expression might be related to activities of transposable elements in embryos.

Our RT-qPCR data suggest that *λ-olt 2-1* is driving *mars2-like* transcription (Fig. 2b). In this case, the LTR of *λ-olt 2-1* might be used as a promoter to allow *mars2-like* expression (Fig. 2b). Several cases have been reported where LTR sequences act as alternative promoters^4^,^8^,^30^. One of the most relevant cases has been reported in mouse embryos at the 2-cell stage, where LTR sequences of MuERV-L regulate stage-specific expression of some protein coding genes^8^. However, in the case of *mars2-like*, it is unlikely that this gives rise to a meaningful protein since many stop codons are found in all reading frames. We therefore assume that *mars2-like* is more likely to be a long non-coding RNA (lncRNA) or a pseudo-gene product. lncRNAs are defined as non-coding RNAs longer than 200 nucleotides. These lncRNAs take on various functions, such as recombination, cell-cell signaling, post-transcriptional regulation, or regulation of chromatin states^31^. The transcript level of conventional *mars2* is strongly reduced from stage 9 to stage 10; at the same time the transcript level of *mars2-like* strongly increases (Fig. 2b). This implies that *mars2-like* RNA might be involved in the regulation of conventional *mars2* expression.

*1a11, λ-olt 2-1* and *xretpos(L)* are marked by H3K9me3 as well as H3K4me3 (Fig. 4a). Our findings show that repression of *λ-olt 2-1* after gastrulation is accompanied by the gain of H3K9me3, whereas the loss of H3K4me3 is not observed in *λ-olt 2-1*. In pre-implantation mouse embryos the repression of retrotransposons (*LINE-1, SINE B2* and the LTR retrotransposon *IAP*) is accompanied by H3K4me3 loss, but not H3K9me3 gain^6^. It was suggested that the loss of H3K4me3 precedes the establishment of heterochromatin via H3K9me3 expansion. In more differentiated cells, H3K9me3 and DNA methylation play a central role in repression of repetitive elements^32^,^33^,^34^,^35^. Interestingly, our results suggest that some of *Xenopus* retrotransposons, especially *λ-olt 2-1*, seem to utilize heterochromatinization for early silencing. This notion is also supported by the appearance of HP1β when transcriptional repression starts (Figs 1b,4c) and by the functional test of H3K9me3 removal by KDM4d (Fig. 4d). The gradual increase in the H3K9me3 level during *Xenopus* embryonic development^36^ (Fig. 4a) may influence such differences between mouse and *Xenopus*. Moreover, differential epigenetic regulation between mouse and *Xenopus* has been reported. For example, *Xenopus* only has very few bivalent histone marks of H3K4me3 and H3K27me3 unlike mice^11^,^12^ and a very low level of H3K27me3 is found in early embryos^36^. H3K9me3 may have a prevalent function to silence genes by promptly recruiting heterochromatin proteins in *Xenopus* embryos.

We have shown an epigenetic mechanism of retrotransposon regulation in *Xenopus laevis* embryonic development. Although expression patterns of retrotransposons have been characterized in many different species, little is known about differential mechanisms of their regulation. It would be an interesting question to ask whether differential retrotransposon regulation affects their activities. Depending on the species, transposon diversity and frequencies of transposition are different^37^. In the case of *Xenopus*, one third of the *Xenopus tropicalis* genome consists of transposable elements, which is comparable to mammalian genomes^38^. However, it shows a high diversity of L1 non-LTR retrotransposons and LTR retrotransposons^38^. Such difference in transposon activities may result from a variety of gene regulation mechanisms for retroelements depending on species. Thus, exploring of the regulation mechanisms of transposable elements will provide insight into development and genomic diversity.

## Methods

### Animals

All experiments using frogs were carried out following requirements of the UK Home Office. All experimental protocols were approved by the UK Home Office.

### RNA extraction, reverse transcription and qPCR

Three oocytes, eggs or embryos per sample were collected and frozen at −80 °C. For RNA extraction, RNeasy Mini Kit (QIAGEN) was used according to the manufacturer’s protocol. RNA was eluted in 35 μl of the provided RNase-free water. For reverse transcription, 12 μl of the RNA solution was mixed with 0.5 μl 100 μM Oligo-dT-Primer and 0.5 μl RNase Inhibitor Murine and incubated at 65 °C for 5 min. All samples were then immediately placed on ice for more than 1 min. Afterwards, 6.6 μl of a reverse transcription mix (4 μl of 5 x First Strand buffer, 1 μl of 0.1 M DTT (dithiothreitol), and 1.6 μl of 10 mM dNTP-mix) were added to the RNA solution. For negative control (RT-), 4 μl of this reaction mix were transferred to a new tube. After adding 0.5 μl of Superscript III Reverse Transcriptase to the reaction mix, all samples were first incubated at 50 °C for 60 min and then at 70 °C for 15 min. All samples were diluted by 7.25-fold with ddH_2_O. For Fig. 1d, gene-specific forward primers (reverse primers as a control) were used for reverse transcription in order to examine whether retroelements are transcribed from both strands. Primer sequences are listed in Supplementary Table S2.

For qPCR, 7300 Real Time PCR System (Applied Biosystems) was used. A reaction mix, consisting of 8.36 μl of ddH_2_O, 0.14 μl of 10 μM Primer mix (forward + reverse), and 12.5 μl of SYBR Green Jump Start Taq Ready Mix for High Throughput QPCR (Sigma-Aldrich) per sample, was used. This reaction mix (21 μl per sample) was then transferred to a 96 Well PCR plate. Afterwards, 4 μl of the template were added. The cycling conditions were used according to the ABI standard: 1 min at 50 °C (x1), 10 min at 95 °C (x1), 15 sec at 95 °C and 1 min at 60 °C (x40). For quantification, a standard curve was created using a serial dilution of embryonic cDNA or, in case of ChIP-samples, of genomic DNA. When we analyzed expression changes of retrotransposons during early embryonic development, values of one-cell stage (stage 1) were set as 1. In some experiments when one-cell stage values are undetectable, the following stage was set as 1.

### Xenopus laevis RNA-Seq

RNA-Seq libraries of *Xenopus laevis* embryos at the gastrula stage were prepared with the TruSeq RNA kit and sequenced on an Illumina HiSeq 2000 instrument as paired ends at 50 base length. This resulted in 88.60 million paired end reads which were filtered for low quality reads (<Q20) followed by trimming of low quality bases from the ends of the reads (<Q20). Reads of good quality where a paired read was of low quality were kept. Adaptors were removed from both pairs using Trim Galore! (based on cutadapt).

### Genome based expression analysis

*Xenopus laevis* genome build 6.1 from Xenbase^15^ was used as a reference genome. Transcript sequence locations on the genome from a previous assembly were used as a background^39^. Repeat and transposable element sequences were obtained by searching the NCBI database for keywords along with *Xenopus laevis*. The resulting sequences were added to the genome and to the junction file for Tophat 2.0.6^40^. Tophat was used to map the RNA-Seq reads to the genome resulting in 77.75 million paired end reads mapped. Read counts were then generated for each of the repeat and transposable elements. These counts were normalized by transcript length and total read counts in order to calculate RPKM values.

### Identification of *mars2-like*

To identify the *mars2-like* transcript a BLAST search was performed, using a RNA-Seq dataset from *Xenopus* embryos at stage 10.5–11.5. The transcript sequence of *mars2-like* was originally found in transcriptome assembly mapped to the *Xenopus laevis* genome 6.1. The *mars2-like* map including exon information shown in Fig. 2a was captured from *Xenopus laevis* 6.1 genome. Moreover, *mars2-like* transcript was amplified using primers binding to exon 1 (Primer sequence: GGACAAGACATGTGAGGAATG) and to exon 2 (Primer sequence: ggccacgcgtTTCTCTACTACCCTTTTCCT or ggccacgcgtAGTAAATAGCATGGAAGAAT). The resulting PCR product was used for DNA sequencing.

### Injection of α-amanitin or KDM4d-encoding mRNA into fertilized embryos

Eggs were *in vitro* fertilized by distributing them in a solution of smashed testis in L15-Medium. After 10 min 5 ml of ddH_2_O was added to the solution and the eggs were incubated for further 20 min. Fertilized eggs were then dejellied in 40 ml of 2% cysteine solution (40 ml of 0.1 x MMR, 0.8 g of Cystein, and 640 μl of 10 M NaOH) for 3 min. The dejellied, fertilized eggs were washed 10 times with 0.1 x MMR (100 mM NaCl, 2 mM KCl, 2 mM CaCl_2_, 1 mM MgCl_2_, and 5 mM HEPES pH 7.4) to carefully remove the cysteine solution. Then stage 1 embryos were transferred to injection medium^41^ (6% Ficoll and 0.4 x MMR) and incubated at 14 °C for at least 15 min. For injection of 4.6 nl of α-amanitin (2 ng or 0.1 ng) or 4.6 nl of KDM4d or myc-GFP encoding mRNA (1 mg/ml), the Drummond Nonoject II Injector was used. Mouse KDM4d was cloned into pCS2 vector and the linearized vector was subjected to mRNA synthesis using MEGAscript SP6 (Life Technology).

### Chromatin immunoprecipitation (ChIP)

ChIP was performed as described before^42^ using 30–60 embryos per sample. To shear chromatin, Biorupter 300 (Diagenode) was used (alternating 30 sec on and 30 sec off for 5 min, 4 times, high output). Prior to ChIP analysis, antibodies against histone H3K9me3 (ab8898, Abcam), against H3K4me3 (ab8580, Abcam) or against H3 (ab1791, Abcam) were conjugated to Dynabeads M-280 Sheep anti-Rabbit IgG (Invitrogen). To do so, 30 μl of Dynabeads were washed three times with 500 μl PBS plus 0.1% BSA at 4 °C. Then 300 μl of PBS plus 0.1% BSA and 1 μl of antibody was added to the beads and incubated at 4 °C overnight.

### Protein extraction and western blotting

For protein extraction, 100 μl of extraction buffer (500 mM Tris pH 6.8, 500 mM NaCl, 1% NP40, 0.1% SDS, 1% β-Mercapthoethanol and protease inhibitor cocktail) was used to homogenize 3 embryos per sample. To separate the resulting suspension, samples were centrifuged for 10 min at 4 °C and 16,000 g. The clear middle layer (80 μl) was transferred to a fresh tube, mixed with 20 μl of 5x loading buffer (0.3 M Tris-HCl, pH 6.8, 50% (v/v) glycerol, 1% (w/v) SDS, 0.05% (w/v) bromphenol blue and 3.2% (v/v) β-mercaptoethanol) and boiled at 100 °C for 5 min. All samples were centrifuged for 3 min at room temperature (16,000 g). The supernatant was then transferred to a new tube.

Polyacrylamid gels (12% SDS) were loaded with 20 μl of this supernatant. Gel electrophoresis and western blots were performed according to standard protocols. For blotting PVDF membranes, a semi-dry transfer system was used (30 min, 25 V). For the protein detection, following primary antibodies were used: anit-H3K9me3 (1:1000, ab8898, Abcam), anti-H4 (1:1000, ab10158, Abcam) and anti HP1β (1:5000, MAB3448, Chemicon). Anti-rabbit or anti-mouse IgG Alexa Fluor 680 (Invitorgen) and/or anti-rabbit IRDye 800CW (LI-COR) were used as secondary antibodies (1:10,000, 1 h at room temperature). The blots were scanned using the imaging system Odyssey (LI-COR).

### Statistical analysis

To test whether there is a statistically significant difference in the mean transcript level, a two-tailed T-test was used. The asterisk is shown if P < 0.05. All error bars represent the standard error of the mean (SEM).

### Primer

All primers used for qPCR are summarized in Supplementary Table S2.

## Additional Information

**Accession codes:** The RNA-seq data have been deposited in NCBI (SRA279316).

**How to cite this article**: Herberg, S. *et al.* Histone H3 lysine 9 trimethylation is required for suppressing the expression of an embryonically activated retrotransposon in *Xenopus laevis*. *Sci. Rep.* **5**, 14236; doi: 10.1038/srep14236 (2015).

## Supplementary Material

Supplementary Information
