# Supplementary material for: Histone H3 lysine 9 trimethylation is required for suppressing the expression of an embryonically activated retrotransposon in Xenopus laevis
Source: Sci Rep. 2015 Sep 21;5:14236. doi: 10.1038/srep14236 (PMC4585706; doi:10.1038/srep14236)
Supplement: Supplementary Information [file srep14236-s1.pdf]

Histone H3 lysine 9 trimethylation is required for suppressing the expression of an embryonically activated retrotransposon in *Xenopus laevis*

Sarah Herberg[1], Angela Simeone[1], Mami Oikawa[1], Jerome Jullien[1], Charles R Bradshaw[1], Marta Teperek[1], John Gurdon[1] and Kei Miyamoto[1,*]

[1]Wellcome Trust/Cancer Research UK Gurdon Institute, University of Cambridge, Tennis Court Road, Cambridge CB2 1QN, UK

*Present address, Laboratory of Molecular Developmental Biology, Graduate School of Biology-Oriented Science and Technology, Kinki University, Wakayama 649-6493, Japan.

Corresponding author: Kei Miyamoto

E-mail: kmiyamo@waka.kindai.ac.jp

Telephone: +81-736-77-0345 (Ext4420), FAX: +81-736-77-4754

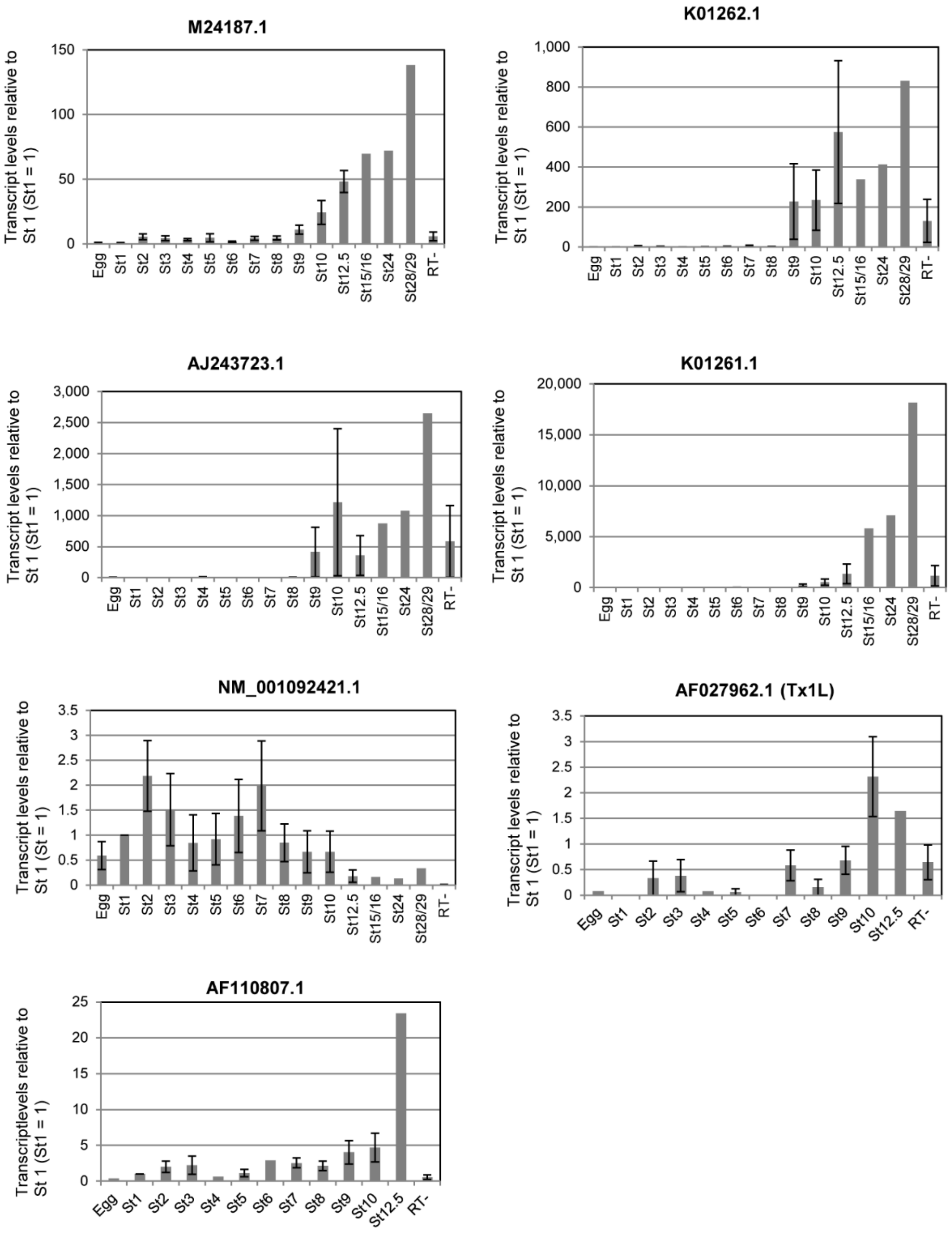

Supplementary Figure S1. Expression analysis of 7 putative retrotransposons in early *Xenopus laevis* embryos by RT-qPCR. Relative changes of the transcript levels of 7 putative retrotransposons during embryogenesis in comparison to their transcript levels at the one-cell stage (St1 = 1). All values were normalized to the overall RNA concentration of the respective sample. All error bars represent SEM. n = 2–5. Sample RT- is a negative control.

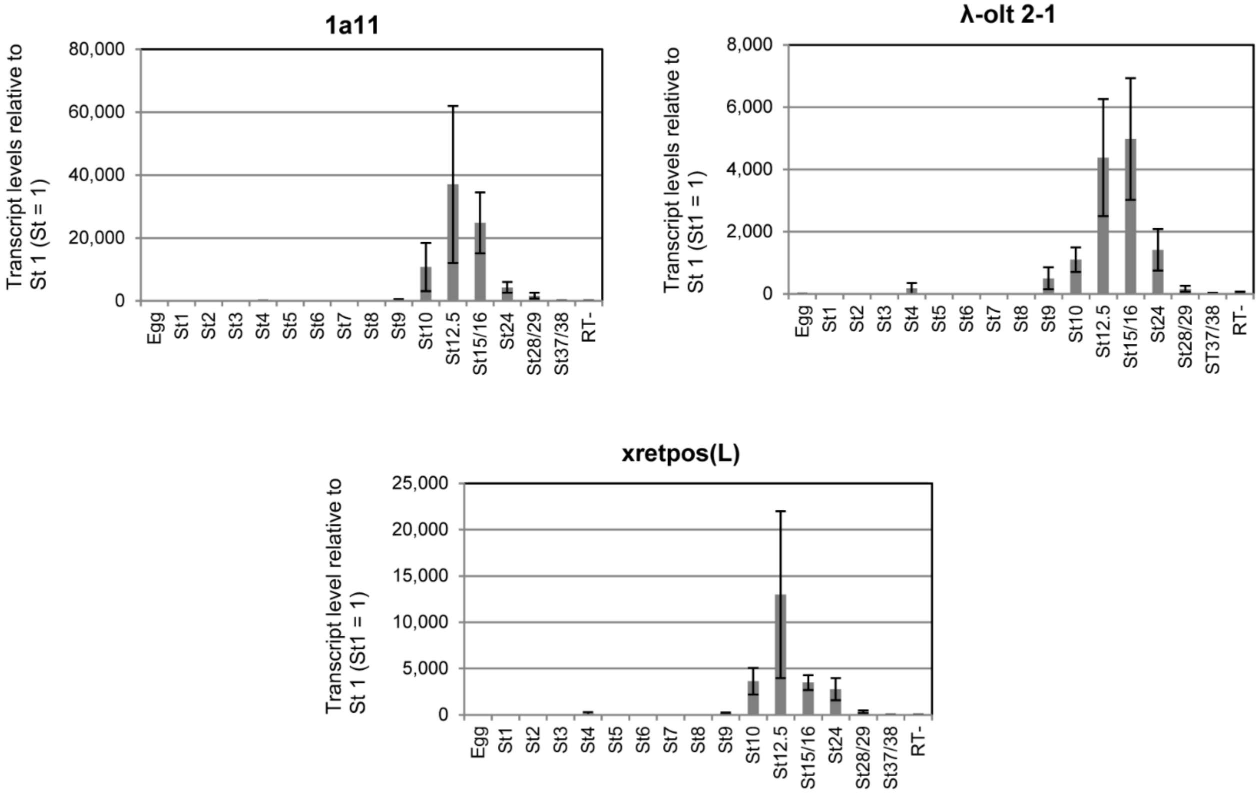

Supplementary Figure S2. Transcript levels of *1a11*, *λ-olt 2-1* and *xretpos(L)* during embryonic development normalized by *pwp1* transcripts. Relative change of the transcript levels of *1a11*, *λ-olt 2-1* and *xretpos(L)* during embryogenesis in comparison to their transcript levels at the one-cell stage (St1 = 1). All values were normalized to the transcript level of *pwp1*. All error bars represent SEM. n = 3–11. Sample RT- is a negative control.

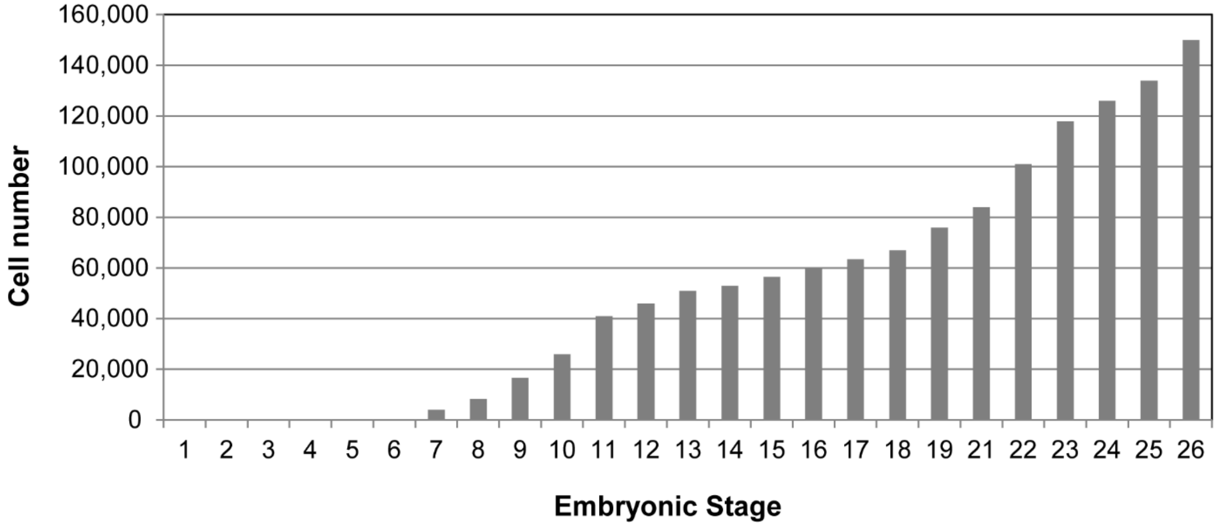

Supplementary Figure S3. Changes in embryonic cell number during development. The total cell number in embryos at different stages was estimated based on DNA concentrations previously reported by I.B. Dawid[43].

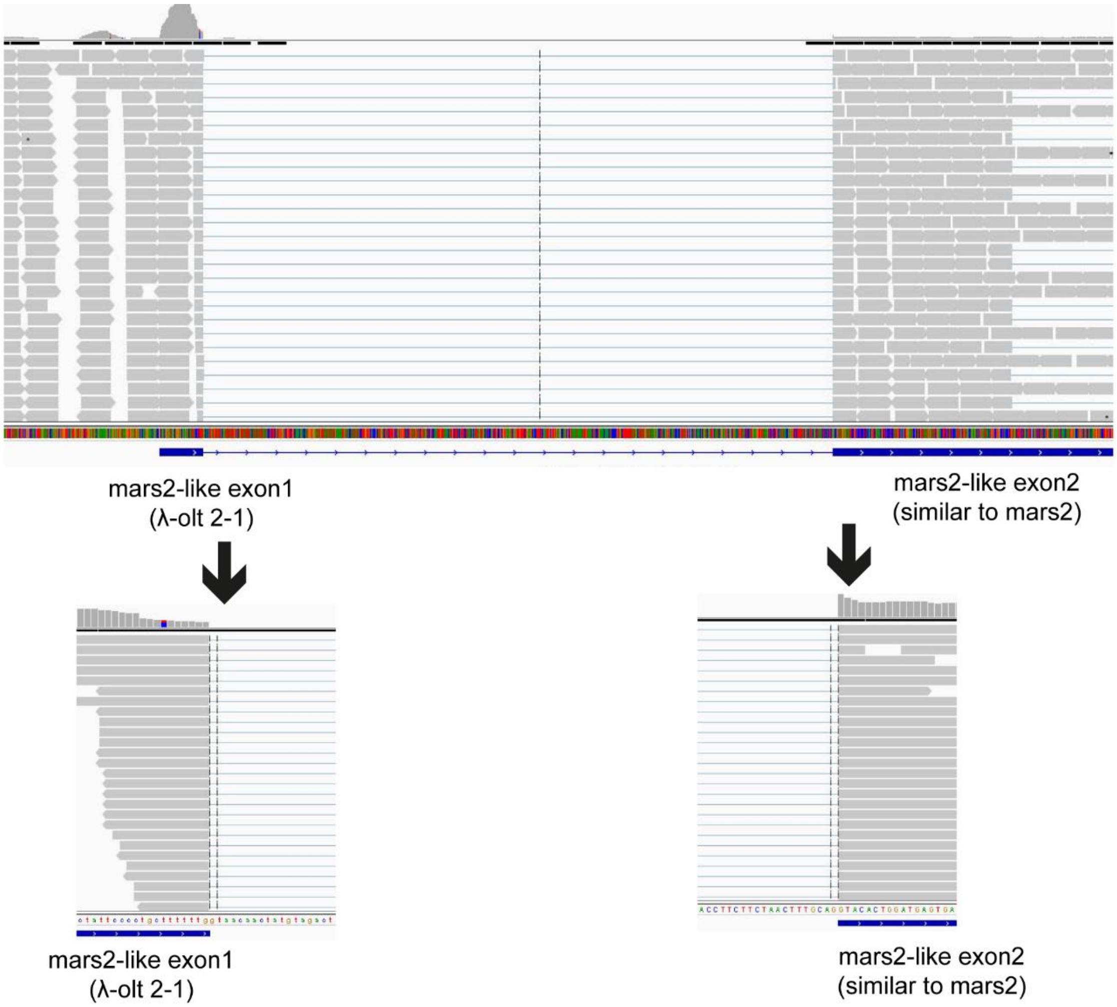

Supplementary Figure S4. RNA-seq reads of *mars2-like* transcript. Paired-end RNA-seq reads from *Xenopus laevis* gastrula embryos were mapped to *Xenopus laevis* genome 6.1. The continuous transcript reads between *λ-olt 2-1* and *mars2*-like sequence were observed. Blue boxes at the bottom represent predicted exons regions. Grey boxes show each sequence reads mapped to the genome.

AGAAATTGCCATCTTGATTTTGTTATTTAATATGAAAGCtgttgttcagggaacttacatgaagccctgattatcataacaaaggttttcctgtgat
tactaaggcctatgtctgctgtggggaccataaaactgattgtgtatgtgagaaaacttgctcaacaggatgtaggcaagttgggggtttatcacag
catcttggcagttgggagggtttctgtgggggcaggtgagaacccaggataaaagaacatgagcagacatgtgaaggagctgggcagtagagaggga
gctgggcagctgagaggaggcagctagagagcgggagaaaccagaggagcctgggacaagacatgtgaggaatgaagaccagaggggaaggcagaga
tgaagccgaactctattcccctgctttttggtaacaactatgtagacttgtgtaatgtgtaactgtaaatattgtaatttttgtttagtctaagtg
taatcatttatgtagaacaatcaattgattattttacaatacatcactttactatacgctcattggtgtggattcattgtttgcttgCTCAAAAGAA
CCAGAAACCCTAGTAAGTGGGTTGAGGTATATTATTGATATTGATATAATATTATTAATATTGATATAATATATATAGAAACTTACACTATTAATAT
ACTTAAACACTTTTTGGGGTTAGTCTTAGCCTCTGCCTTATTGTGCACCTCATTTTCTATATATGCCTTCTGGATTGCTGTTTTACAACATTTGTTA
TAGTGTTTATATTCAGTAAATGCATCTTCTGTCCCCACAGACTTGTAGTTTTTACATAAACTATTTTGTTCCTTTCTTACATTTTTACACTATTATA
AACTGCTAATTTAATGAATTTTGTAGCAACTTCACTAGCTGCTGTTGCTCTGCTCAGAGTAGAAAAAGGTAATCTGAGGTTTTTGGTCTATTTCCTG
GGCAGAGCTACAGCTCTGTTTTCCTTCTGAACATGTTTGACTGTGTAGTAGAAAAGTGACCAACAGGCAGTGCTGTTACAAAATTAAAAATAAATAA
TTTGGTTAAAACAGCTTAATACCTTTGAAACAAACAAAAACAACAGTGCCACACCAGCTTTGCAGCTTTGTTTTTTTATATTAAGATTGAAACAATG
TACTATGTGTATCAAATAAGAAAGCAAGAGCCCTGTAACAATGAAGATCGCTAAAACTCCCTATCCTGATTGAATTTGCCTGCTGTACTACTGACAA
ACACCTGCCAAGAGTACAGGCATTTTGCCAGATATACCAGGGAAAAGGTGACCAGGGTCTTGCAGACAATTGTTCTACATTTCTGCACAGTATGTAC
AAAGTGTATTGATACTTAATAGGCCTACTATGCCATGGGTCAAGTATAGTACATCCTGTAGCATGATCCTTCTTTATTATTTAGCTGATTTCAATAT
TACTGAACCTGTAAACACCTTCTTCTAACTTTGCAGGTACACTGGATGAGTGAGGAGAACTACATTTTCACCTGTCATCTCTTCACCCTGCTCTGCT
AAACTGGTTACAGATAGAACCTGTGCATCCTTCTCCATTCCTTAAATTAGTACATCACTGGCTCGAGGAAGAAGTCCCAGACCATTCAGTGTCTAGA
CATTGCAGTAGTTTATCCTGGGGAATCCCTGTGCCTTCCGATGCCTCTCACATTATATTTGTCTGGTTAGATGCTCTAGTAAAGCAACTGCTGCTGG
ATATCTTAAGCCATCGTTGGCACTGTGGGGCCCTTCCACCCACCTCCTTGGTAAGGACATTTTATTCTTCCATGCTATTTACTGGCCTGACTGCTTC
TTCATTCCCATTGGACTTCAGAAGGTACAAAAATGTCTAAAAGCCTAAAAAATGTGGTGAATCCCTCAGATTGCATCAGGCGTTAAACAAAAGTTGG
GCTGCACTACCATTTGATTTGCCATGGTGCACCAGAGAGATACTGTAATTTCACACACCGTACTACCCACATGCTGTTGAACTCAGAGTTAGCTGAC
CCACTTGGGGGGTCTGCTAAACTGTTGCACTGCACCTGCTATCAACCCAATGCAACACTTTACAAAATTCCACTATGACAGTTTTCCTTTAGCTTTC
TGTGACCAGATACATGATTTGCTTGCTGCTTTGCAGGAACTCCCTGCAGAGATGGACCAATGGATAAAGAAATTTCAAGTTCACAAGGCTTTGGAGT
GTATGGATGCCTGTGTACGCTGCTCCAATGCCTTCTTCCAGACCCAGGCTCCTTGAAAAGGAAAAGGGTAGTAGAGAAAGAGGCTGCTTTGAGGGAC
TCTGTTATATACCTGACATTGGAAGCTCTTCGACTGTATGCCACACTTCTTCATCCTGCAGTGCTTGGATTGGCTACAGCGGCACTAGACAGGCTTG
GTGTGCCCCTAAATATGAGGACGCTTATGAGGAATACATTCTTAGCAGCTACAAGAGGATAAATTTGTTACTTTCGGGGTCAAACTTTAGGTCAGGA
CAAAGACCTGCTCTTTCCTAGGTTGGAGAAGAGTAAAGCTTTATAGTAAGAAGACTGCTGGAGTTAGCCATAAAGGTCACATAGGGATATATTCAAA
TATTAATGTCTCCCCCAATCTGTTATTGATGTTTACCTTTTATGTTGGTTTATGTTAAGGCACCTCATCCTAATCTCATTTGAAATCTTTTTCTTTG
ATGAAGAAAAACATAATTAAAATGTAATTGAAAGACTCTAATGTTCTATAGCATATTAGGCAAATGTTATTGTTTTTCTCTCATATGACAATGCTTT
TTTAAGGGACCAGTAACGTCAAAATTTTTTTTTACAAAATTCGTTAGTATACATCGNNNNNNNNNNNNNNNNNNNNNNNNNNNNNNNNNNNNNNNNN
NNNNNNNNNNNNNNNNNNNNNNNNNNNNNNNNNNNNNNNNNNNNNNNNNNNNNNNNNNNNNNNNNNNNNNNNNNNNNNNNNNNNNNNNNNNNNNNNN
NNNNNNNNNNNNNNNNNNNNNNNNNNNNNNNNNNNNNNNNNNNNNNNNNNNNNNNNNNNNNNNNNNNNNNNNNNNNNNNNNNNNNNNNNNNNNNNNN
NNNNNNNNNNNNNNNNNNNNNNNNNNNNNNNNNNNNNNNNNNNNNNNNNNNNNNNNNNNNNNNNNNNNNNNNNNNNNNNNNNNNNNNNNNNNNNNNN
NNNNNNNNNNNNNNNNNNNNNNNNNNNNNNNNNNNNNNNNNNNNNNNNNNNNNNNNNNNNNNNNNNNNNNNNNNNNNNNNNNNNNNNNNNNNNNNNN
NNNNNNNNNNNNNNNNNNNNNNNNNNNNNNNNNNNNNNNNNNNNNNNNNNNNNNNNNNNNNNNNNNNNNNNNNNNNNNNNNNNNNNNNNNNNNNNNN
NNNNNNNNNNNNNNNNNNNNNNNNNNNNNNNNNNNNNNNNNNNNNNNNNNNNNNNNNNNNNNNNNNNNNNNNNNNNNNNNNNNNNNNNNNNNNNNNN
NNNNNNNNNNNNNNNNNNNNNNNNNNNNNNNNNNNNNNNNNNNNNNNNNNNNNNNNNNNNNNNNNNNNNNNNNNNNNNNNNNNNNNNNNNNNNNNNN
NNNNNNNNNNNNNNNNNNNNNNNNNNNNNNNNNNNNNNNNNNNNNNNNNNNNNNNNNNNNNNNNNNNNNNNNNNNNNNNNNNNNNNNNNNNNNNNNN
NNNNNNNNNNNNNNNNNNNNNNNNNNNNNNNNNNNNNNNNNNNNNNNNNNNNNNNNNNNNNNNNNNNNNNNNNNNNNNNNNNNNNNNNNNNNNNNNN
NNNNNNNNNNNNNNNNNNNNNNNNNNNNNNNNNNNNNNNNNNNNNNNNNNNNNNNNNNNNNNNNNNNNNNNNNNNNNNNNNNNNNNNNNNNNNNNNN
NNNNNNNNNNNNNNNNNNNNNNNNNNNNNNNNNNNNNNNNNNNNNNNNNNNNNNNNNNNNNNNNNNNNNNNNNNNNNNNNNNNNNNATACTAACGAATTTT
GTAAAAAAAAATGTTGACGTTACTGGTCCTTTAAGGAGAACTAATCCCAACCCTAAGAGACAGTCTCCATCCAGTAGCCCCCATCCGCCCCCCTCTA
TACTTCTTCGCTGCTTAGTCCATTATTTGTAAAAGTGTTGCAGAAATTTGCACACACCTCTGCATGGAGATGCAGAGTAGCACAATGGAGCTCACGG
ATGCCATCTTTTGCATACATTCACTAAAGGACATCCAACACCAAGCTTTTTTGCGCATGTGCAGTTGGAGCTATTTGGTATATGCTCCTACTGTGCA
TGCACTGGAATGCTCCGTAAATAGCCGAAGTGAAGAAGAGGATCTGAAGATGGCACCCATGAGCTCTGCTGCACTACTCTGCATAGAGAGGTCTGTG
CAACTTTCTAAATAGTGGACAATGCAGGTAGGGGACAGGTGGGGCTTTCAGATGCAGACTCTCTTTAGGAGGGGTGTTTAGTTCTCTTTTGAAAAAA
AATTGACGTGTTTCTTTGAAAATCTATAGGAAACCAGTATTACTAGACTACAATATAGATTGAAAAGTTTTCCCAAAACCAGCCAACCTTGTATAAT
GATTTAGACAGAGTGCAGCAGATAATCTTTCTTTGGGATATCATGCAGTGCAGGAGTATCGGCTATGGAATGTACGGTAATTATTATACGGTATAGT
AATTATTGCTAGGCCAGTGGAGGATAGTCCATTTCCATTCTTTTTAGAAATATTGTCACATTCCTATGTTTTTTCATACAATCATGGCAATAATGCT
ACATGTATTCTATTTCTTTACAATGTTTTTTTATGGATATTTGTCAAAACAACTTCATCACAAGGTGTATTCTGTTTATTAATAGAAGTGGAATACA
GGCATGAGATCTGTTATCCAGAATGCTTGGGACCTGGGG

Supplementary Figure S5. Nucleotide sequence of a *mars2-like* transcript. The dark blue marks represent exons as partially validated by reads from RNA-seq. Exon 1 and the light blue marks represent a cluster of reads that shows 97% sequence identity to the LTR of *λ-olt 2-1*. Exon junction between *λ-olt 2*-1 and *mars2* homology sequence was further confirmed by sequencing. N represents non-determined sequences.

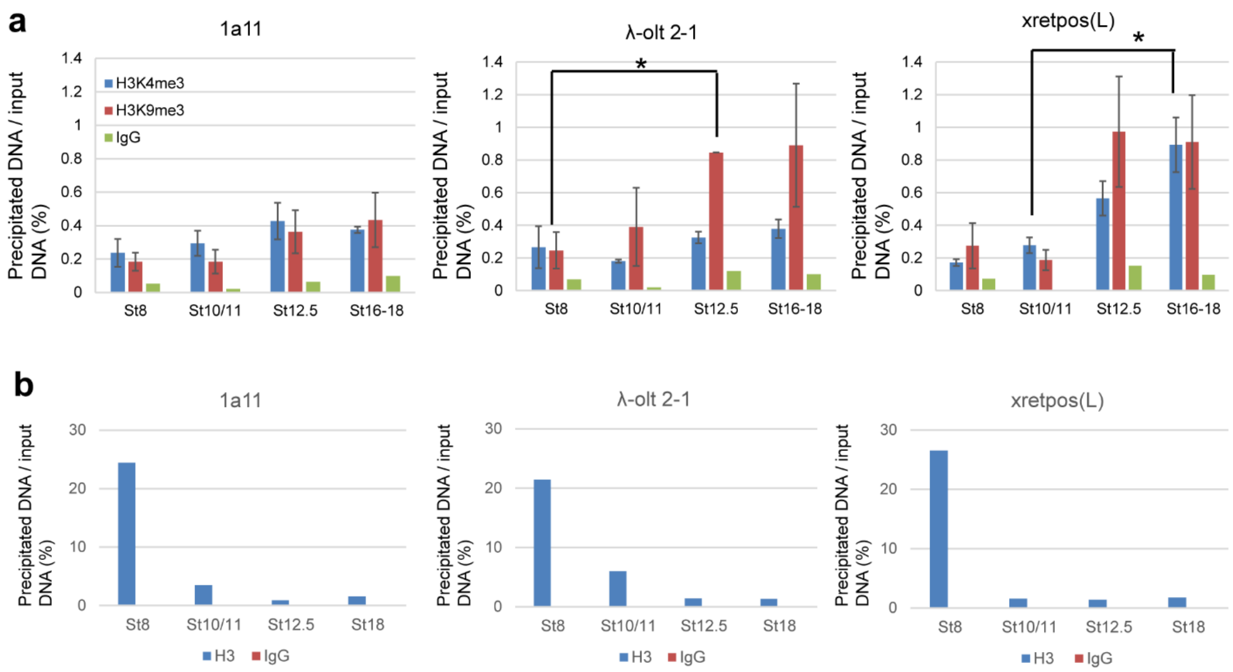

Supplementary Figure S6. H3K4me3 and H3K9me3 are enriched on LTRs of retrotransposons. (**a**) ChIP analysis indicates enrichment of H3K4me3 and H3K9me3 on LTRs of retrotransposons at different stages of embryogenesis. A significant increase of H3K9me3 signals in *λ-olt 2-1* and that of H3K4me3 in *xretpos(L)* were observed. Y-axis represents the precipitated DNA/input DNA of *1a11, λ-olt 2-1* and *xretpos(L)*, as determined by qPCR. (H3K4me3 and H3K9me3: n = 3–4, IgG: n = 2). All error bars represent SEM. $*P < 0.05$. Blue = H3K4me3, red = H3K9me3, and green = IgG. IgG was used as a negative control. (**b**) Histone H3 was used as a positive control for ChIP experiments using embryo samples. H3 signals are higher in early embryonic stages, which might reflect chromatin with packed nucleosomes before midblastula transition. (H3: n = 1–2, IgG: n = 2).

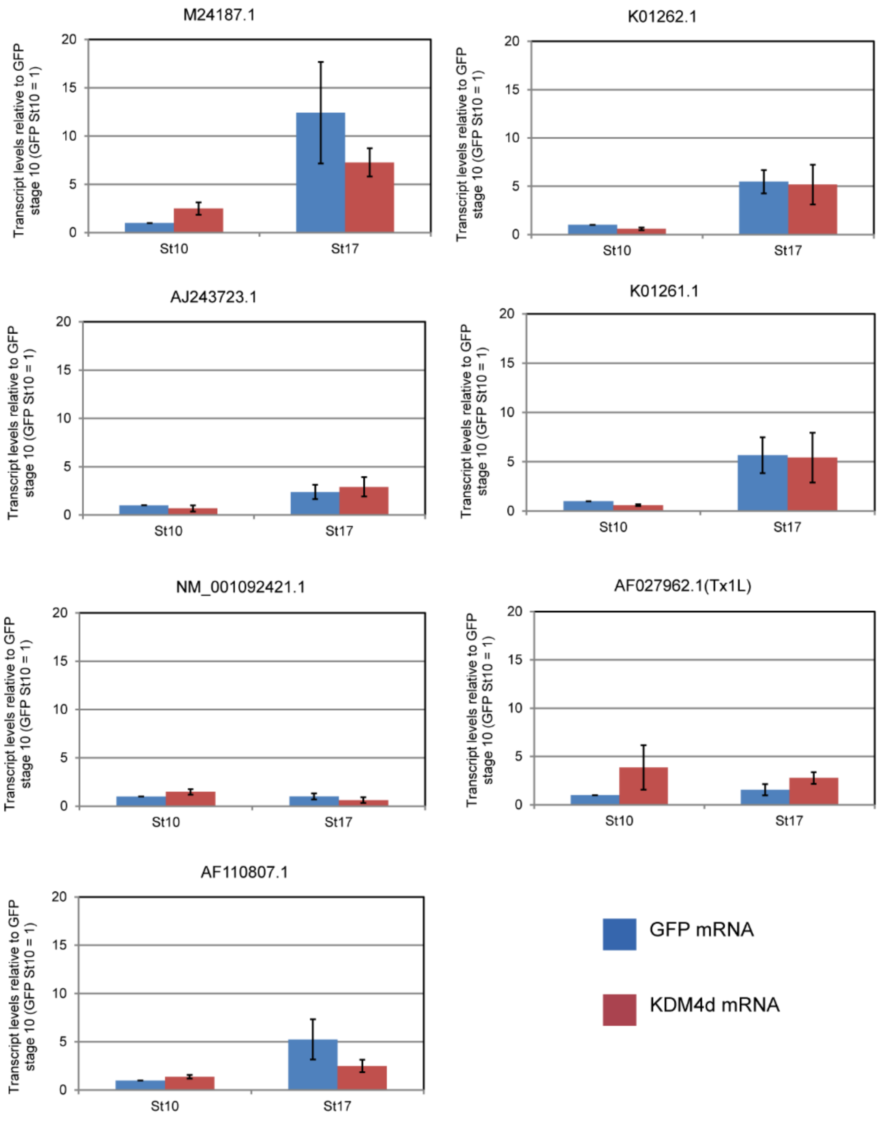

Supplementary Figure S7. The effect of KDM4d overexpression on the expression of putative retrotransposons shown in supplementary Figure S1. Putative retrotransposons expression was measured by RT-qPCR at the gastrula (St10) and the neurula stages (St17). The relative change of the transcript level after KDM4d overexpression in comparison to the transcript level of control myc-GFP-mRNA injected embryos is shown. The transcript level of myc-GFP-mRNA injected embryos at stage 10 was set as 1. All values were normalized to the RNA concentration of the sample. No statistically significant differences were detected although AF027962.1 transcription seems slightly upregulated after KDM4d overexpression. All error bars represent SEM. n = 3. Blue bars represent myc-GFP mRNA-injected embryos while red bars are KDM4d mRNA-injected embryos.

Supplementary Table S1: Retrotransposons used for expression analysis in early *Xenopus laevis* embryos (related to Supplementary Figure S1).

NCBI_ID	Gene description	RPKM values	Analyzed by RT-qPCR
L11263.1	1a11	201189.674	Yes
AF145965.1	λ-olt 2-1	109503.33	Yes
AF057166.1	xretpos(L)	171488.054	Yes
K01261.1	PTR-1 repeat	46458.5436	Yes
K01262.1	PTR-2 repeat	66688.4882	Yes
AF027962.1	retrotransposon L1Xl ORF1 and ORF2, CR1 ORF2-like, and LTR ORF2 pseudogenes, complete sequence; and retrotransposon Tx1L, partial	5422.76883	Yes
AJ243723.1	proviral gypsy retrotransposon partial reverse transcriptase and protease genes (pol gene)	743.907942	Yes
NM_001092421.1	gypsy retrotransposon integrase 1 (gin1)	1432.74362	Yes
M24187.1	unknown protein mRNA, partial cds, and repetitive element, 5' end	7868.18117	Yes
AF110807.1	Frt628 repeat region	3249.09543	Yes
AF145966.1	Highly similar to xretpos(L)	121075.062	No
AJ506107.1	Xen1	49.3789629	No
M26915.1	Tx1	3279.44805	No
NC_010955.1	endogenous retrovirus Xen1, complete genome	43.0074838	No
S80540.1	Ocr repeat region	7374.75975	No
X00077.1	repetitive 1723 element fragment C17	17186.3414	No
X00078.1	repetitive 1723 element left terminus sequence	13506.5099	No
X00079.1	repetitive 1723 element right terminus sequence	12193.8163	No

Supplementary Table S2: Primers used for qPCR and ChIP qPCR.

Primer name	Sequence (5'-3')	Analysis
AF027962.1 F	CCAGAGTAACACGGTGGTTCCT	qPCR
AF027962.1 R	AATGGACTCGGGAAAGAGCAT	qPCR
AF057166.1 (xretpos(L)) F	ACCTTAGTAGTCACAGGAAAACCTTTG	qPCR
AF057166.1 (xretpos(L)) R	AAGCGGTTGTTCAGGGAACTT	qPCR
AF110807.1 F	TCCCATGTGCCCCCTTAA	qPCR
AF110807.1 R	CTAGTTCCTGCTTTTCAGCTCTCTAAC	qPCR
AF145965.1 (λ-olt 2-1) F	GATTATGTGGCCAGGGCAAT	qPCR
AF145965.1 (λ-olt 2-1) R	CCAGTGCACCTTTACCCTTTG	qPCR
AJ243723.1 F	CCCCACACCGGCAGTATG	qPCR
AJ243723.1 R	GAGGAGGAGTGGAACCAGGAA	qPCR
mars2-like (Primer 1) F	TGCTTTTTTGGTACACTGGATGA	qPCR
mars2-like (Primer 1) R	GCAGAGCAGGGTGAAGAGATG	qPCR
Conventional *mars2* (Primer 2) F	CCCCCACCTTGGACATGTAT	qPCR
Conventional *mars2* (Primer 2) R	GCGGAGTAGCGATGTTGCA	qPCR
DQ096947.1 (1a11) F	CGTGTGAGTTCTGCACAGGTTAG	qPCR
DQ096947.1 (1a11) R	CGTGACACAGCATGCAACTG	qPCR
K01261.1 F	ACAAACAGTCCCGTGGGTAACT	qPCR
K01261.1 R	CCCCACAAAAATGCAGTCAA	qPCR
K01262.1 F	CCCCATTATATGCCCCACATT	qPCR
K01262.1 R	CCCTGGCGTTCATATTTAGGAT	qPCR
M24187.1 F	AGGGTGGTTGTTAATGGGACAT	qPCR
M24187.1 R	GCCCTGAGGAACCCCACTAA	qPCR
NM_001092421.1 F	CGACCCCATGGTCTCTCAGA	qPCR
NM_001092421.1 R	CCTCGCATAACATGAACCTTAGC	qPCR
pwp1 F	GGGACTTTCACCATTGACTTAAACA	qPCR
pwp1 R	GACTTCGAAAATCTGGCATCTCA	qPCR
Sox17a F	CGTCCTGGGCTGGAGATGT	qPCR
Sox17a R	TCTCCTCTGGATTTGGCAGAA	qPCR
1a11-LTR F	CCTTTGACCTGAAGAGAAGTGTGTAT	ChIP qPCR

1a11-LTR R	CAACGTCCCGTCTAACTAGCAA	ChIP qPCR
xretpos(L)-LTR F	CAGAGGAGCCTGGGACAAGA	ChIP qPCR
xretpos(L)-LTR R	GGGAATAGAGTTCGGCTTCATCT	ChIP qPCR
λ-olt 2-1-LTR F	AAGCTGTTGTTCAGGGAACTTACA	ChIP qPCR
λ-olt 2-1-LTR R	GGCCTTAGTAATCACAGGAAAACC	ChIP qPCR

Supplementary reference

43 Dawid, I. B. Deoxyribonucleic acid in amphibian eggs. *J. Mol. Biol.* **12**, 581-599 (1965).
